# Evolution of branched peptides as novel biomaterials

**DOI:** 10.1039/d4tb01897d

**Published:** 2025-01-13

**Authors:** Matthew J. Little, Jody M. Mason, Nazia Mehrban

**Affiliations:** a University of Bath, Claverton Down Bath BA2 7AY UK nm2051@bath.ac.uk

## Abstract

Branched peptide-based materials draw inspiration from dendritic structures to emulate the complex architecture of native tissues, aiming to enhance the performance of biomaterials in medical applications. These innovative materials benefit from several key features: they exhibit slower degradation rates, greater stiffness, and the ability to self-assemble. These properties are crucial for maintaining the structural integrity and functionality of the materials over time. By integrating bioactive peptides and natural polymers within their branched frameworks, these materials offer modularity and tunability and can accommodate a range of mechanical properties, degradation rates, and biological functions making them suitable for biomedical applications, including drug delivery systems, wound healing scaffolds, and tissue engineering constructs. In drug delivery, these materials can be engineered to release therapeutic agents in a controlled manner, enhancing the efficacy and safety of treatments. In wound healing, they provide a supportive environment which promotes rapid and efficient tissue repair. The combination of biomimetic design and functional adaptability makes branched peptide-based materials a promising candidate for the development of next-generation biomaterials, paving the way for significant advancements in healthcare.

## Abstract 2

Branched peptide-based systems mimic dendritic structures to improve biomaterial performance for medical applications with slower degradation, greater stiffness, and self-assembly capabilities.

## Graphical abstract

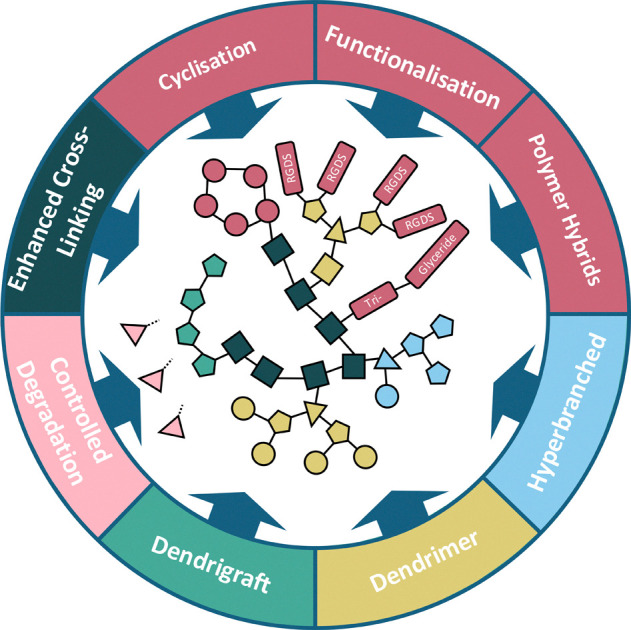

## 1. Introduction

Biomaterials have progressed significantly over the last century, transitioning from simple systems replicating tissue mechanics, such as titanium alloys^1^ and calcium-phosphate ceramics,^2^ to biologically active materials that mimic native tissue chemistry and architecture, enhance repair, or prevent immunological rejection, such as collagen^3^ and gelatin methacryloyl (GelMA).^4^ Of note is the shift from inert materials to biologically functional systems capable of interacting with cells and promoting regeneration.^5–9^ Polymers are particularly attractive due to their ease of synthesis and flexibility for molecular modification.^6^ Polymers encompass a wide range of natural and synthetic materials, each with distinct physical and chemical characteristics.^10,11^ Synthetic polymers, such as polyethylene glycol (PEG), are hydrophilic and scalable^12,13^ but biologically inert without chemical modification.^14^ In contrast, natural proteins, like collagen, offer cellular compatibility by preserving amino acid sequences, such as RGDS, recognised by cell membrane integrin receptors like αVβ5.^15^ Natural proteins, however, suffer from limitations in structural integrity and batch-to-batch consistency.^16^ Peptides, particularly branched peptides, offer a compelling alternative combining the bioactivity of natural proteins with the tunability and reproducibility of synthetic systems.

Peptides are short chains of amino acids that can be synthesised and modified to control their chemical and physical properties, including charge, isoelectric point, and solubility, as well as their three-dimensional (3D) assembly,^17,18^ stiffness,^19^ and porosity.^20,21^ When used *in vitro*, these alterations can dictate specific cellular responses, such as proliferation,^22^ differentiation^8^ and migration^23^ which in turn support advancements in biomaterial design, including tissue regeneration.^24–29^ Unlike linear peptides, branched peptides introduce branching points at amide or carboxyl side chains, enabling diverse structural architectures and functionalities such as self-assembly,^30^ bio-active functionalisation,^7,31,32^ and the ability to act as drug carriers.^33^ These branching points provide unique opportunities for tailoring mechanical stiffness,^7,30^ fibril diameter,^32^ and biodegradation rates^34^ to meet specific biomedical needs. Novel and updated methods of synthesis have further decreased the cost and expertise required for their development, which previously hindered progress.^35–37^

**Fig. 1:**
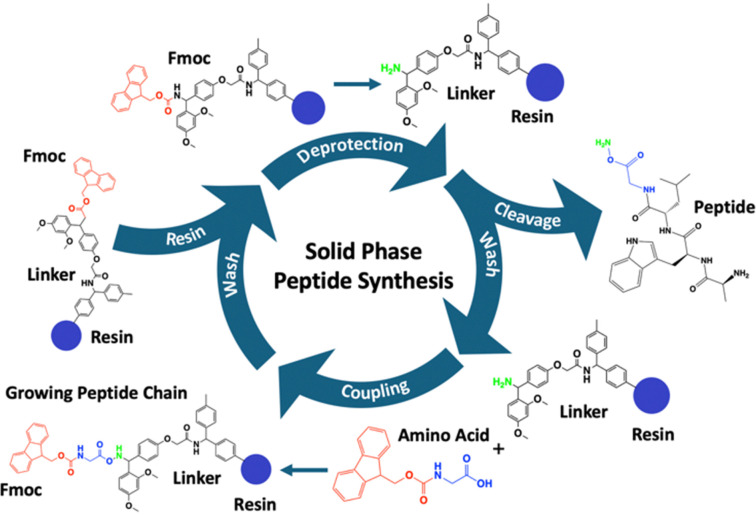
The key steps of solid-phase peptide synthesis (SPPS). The resin is first saturated in an organic polar solvent such as *N*,*N*-dimethylformamide (DMF) or dichloromethane (DCM). This is followed by deprotection of the Fmoc group to expose the amino group for subsequent coupling reactions. Washing steps to remove unbound reagents and by-products, ensure that the resin is free of contaminants. Amino acid coupling then follows, facilitated by activating agents, to attach the next amino acid in the sequence. This is followed by additional washing to maintain reaction fidelity. Finally, cleavage of the synthesised peptide from the resin is performed, along with sidechain deprotection, to release the final peptide product.

A key enabling technology for the development of peptide-based biomaterials is solid-phase peptide synthesis (SPPS), a method introduced by Merrifield in 1963 which revolutionised peptide chemistry.^38^ SPPS involves the sequential addition of amino acids to a growing peptide chain formed *via* a condensation reaction between amines and carboxylic acids anchored to an insoluble resin (Fig. 1).^38^ This approach enables precise control over peptide sequence and allows for the incorporation of non-natural amino acids, functional groups, and branching points with high efficiency and purity.^39^ SPPS chemistry relies on the activated ester from the incoming amino acid attacking the amine of the amino acid bound to the resin. Following the same SPPS principles, the process can be applied to the amine side chain on lysine, enabling the creation of branching points. To utilise the amine side chain, SPPS employs orthogonal protecting groups, including; 4-methyltrityl (MTT),^40^ allyloxycarbonyl (Alloc),^41^ and 1-(4,4-dimethyl-2,6-dioxocyclohex-1-ylidene)-3-methylbutyl (ivDdE),^42^ which can be selectively removed under specific conditions. Protecting groups such as these provide selective functionalisation and the incorporation of branching points. Alternatively, the carboxylic acid side chains on aspartic acid and glutamic acid can also be used as branching points although the orientation is reversed. The activated ester is bound to the resin, and the amine will be the incoming amino acid, typically using the Alloc protecting group^41^ or bis(2-sulfanylethyl)amido (SEA) *via* native peptide ligation.^43^ The use of orthogonal protecting groups allows the synthesis of complex architectures, such as dendrimers and multi-arm peptides, which are challenging to achieve through other methods such as liquid phase peptide synthesis.^44^ Furthermore, SPPS facilitates the incorporation of novel amino acid sequences, functional groups or bioactive motifs, enabling precise tuning of peptide properties for applications such as self-assembly, extracellular matrix (ECM) mimicry, or drug delivery.

The composition of amino acids in the peptide-based material can directly dictate cellular behaviour. Self-assembling peptides utilising combinations of polar and non-polar amino acids to direct folding, tangling, can be used to create biomaterials. By utilising the natural secondary structures, α-helices and β-sheets, peptides can support the propagation of self-assembling 3D materials such as hSAF (hydrogelating self-assembly fibres)^8,17^ and RADA16-I systems.^45^ α-Helices require specific polar and non-polar regions in the peptide sequence to create turns in the linear chain, whereas β-sheets utilise alternating polar and non-polar amino acids to create large folds, forming stacked sheets with a myriad of examples utilising them to create 3D materials.^8,17,46,47^ The creation of 3D secondary structures, whether α-helical or β-sheet based, allows these materials to more closely mimic fibril structures typically seen in the ECM. The dynamic fibrous environment of the ECM influences cell signalling transduction, as well as degradation and reconstruction of tissue matrices.^48^ Mimicking the ECM is an ideal way of mitigating a negative immunological response to biomaterials and influencing cell–cell paracrine signalling to promote acceptance by the body. This strategy has further been shown to improve damaged ECM reconstruction.^49–51^ Other functional moieties can also be appended to a peptide chain to increase cellular compatibility.

Functionalisation of peptides or polymers aims to chemically couple additional moieties to improve for example, the biological properties of materials, such as targeting the cell receptors/integrins which control cellular response to environmental stimuli; crucial for tissue repair, immune response, and development, such as cell survival and proliferation.^52,53^ A popularly used moiety for material functionalisation is the RGDS peptide. This sequence is repeated in many proteins, for example fibrinogen, and can interact directly with integrin αVβ5, promoting cell adhesion and proliferation, and contributing to biomaterial integration with cells both *in vitro* and *in vivo*.^54–57^ Functionalisation is achieved by chemical ligation to modified side chains through reactions such as Cu-catalysed cycloaddition (CuAAC),^58^ strain-promoted azide–alkyne cycloaddition (SPAAC),^59^ thiol–ene,^60^ Staudinger ligation,^61^ and others.^62^

Given their versatile properties, branched peptides are underrepresented in biomaterial research. This review introduces branched peptides and provides an update in this field, such as novel orthogonal deprotection methods, functionalisation with bioactive peptides, such as RGDS and IKVAV and other long-chain polymers, including lipids and carbohydrates. We also review the current state of supramolecular self-assembly using branched α-helices and β-sheets focussing on how these structures may be adapted to different solvent environments, particularly for modifying material stiffness and degradation rates and the limitations associated with current experimental designs. Finally, we outline how branched peptide research has influenced other fields including oncology and infectious diseases, and discuss the future of branched peptide-based biomaterials.

## 2. Branched peptide design, structure, and synthesis

### 2.1 Overview of branched peptides

Early iterations of branched peptides were synthesised using thermal homo- and co-polymerisation, primarily of lysine, glutamic acid and aspartic acid,^63^ creating arbitrary structures with limited applications.^35,64^ Current peptide syntheses rely heavily on SPPS, rather than liquid-phase peptide synthesis (LPPS) offering greater chemical stability by controlled fluorenylmethyloxycarbonyl (Fmoc) deprotection, efficient couplings, and a variety of side chain protecting groups, removable under conditions such as *tert*-butyloxycarbonyl (Boc or *t*Bu) in 50% trifluoroacetic acid (TFA) and 2-acetyldimedone (Dde) in hydrazine.^65^

**Fig. 2:**
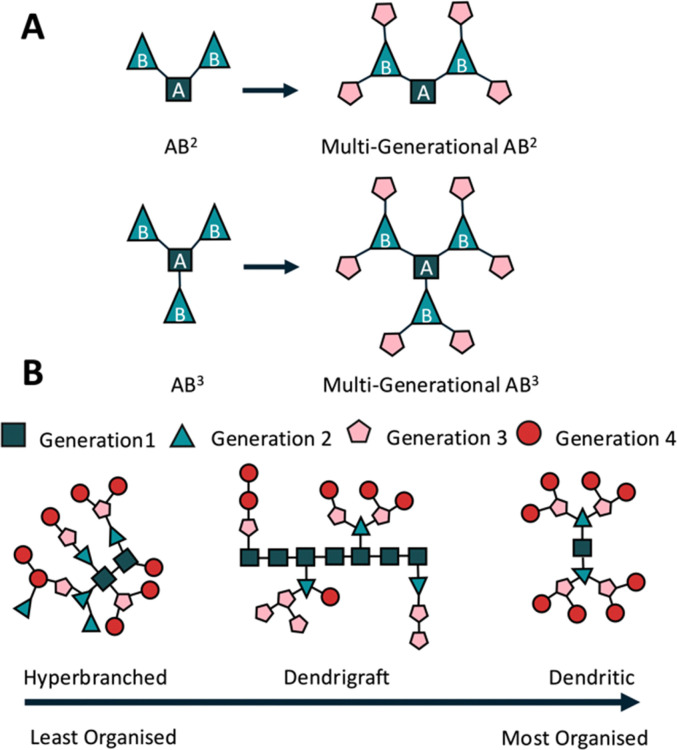
Polymer branching types. (A) AB^2^ & AB^3^ polymer branching types, dictating the formation of either two bonds (AB^2^) or three (AB^3^) from a starting amino acid. (B) Peptide branching conformations, hyperbranched peptides represent the least organised structure, synthesised through a one-pot synthesis originally using thermopolymerisation, forming complex 3D networks, but with an arbitrary structure. Dendrigraft peptides are typically formed through coupling of linear, or dendritic peptides, onto a linear chain, representing a relatively organised and most recently developed of all three branched peptide types. Dendritic peptides are synthesised through sequential couplings and deprotections to create stable structures (typically used as drug delivery vectors).

Most branched peptides can be separated into three broad categories based largely on the AB^2^ branching method, where the starting amino acid (A) can form two branches (B^2^)^37,66^ (Fig. 2A). Hyperbranched peptides are highly branched, 3D structures with multiple functional end groups (Fig. 2B). They are synthesised through global deprotection and one-pot coupling reactions,^67^ where, due to the limits of SPPS and steric hindrance, some branches may be longer, others shorter, and some non-existent; a problem faced by most hyperbranched polymer systems.^67,68^ This creates the least organised of all three structures but does make them suitable for exploring extended molecular interactions and scalable syntheses.

Dendrimer peptides are tree-like macromolecules with a central core and multiple branched generations (Fig. 2B). They provide precise control over size, shape, and functionality and are ideal for gene delivery^69^ and bioimaging.^70^ Tam first described using the lysine core to synthesise a peptide dendrimer as an antigen presenter for vaccine design and multiple antigen peptides (MAPs).^71^ The polyvalency is generated by multiple outward-facing arms, allowing them to be used as drug carriers.^72^ The addition of natural peptide functionalities reduces targeted immunological events, thereby increasing peptide half-life *in vivo*.^73^ The typical radial shape also reduces enzymatic contact with molecules closer to the centre, reducing degradation and allowing them to be used as drug vectors.^74^

Dendrigraft peptides present a linear peptide in their centre, branching within the chain or at the termini^66^ (Fig. 2B). These branches may be other linear^30^ or dendritic structures,^75^ such as the dendrimer peptides described earlier. Dendrigraft synthesis relies on the coupling of peptide chains, as opposed to the single amino acid couplings used for dendrimer synthesis. This increases the ease of synthesis and promotes bulk multi-gram scale production.^76^ Using alternative side chain deprotection allows for multiple peptide couplings without compromising other side chains and branching points, enhancing adaptability and control over functionality. Multiple deprotection strategies, SPPS chemistry, and fewer branching points compared with the two previously described branched peptide systems improves dendrigraft synthesis, making them easier to manipulate and offering controllable functionality and degradation rates.^30^

### 2.2 Methods for branched peptide synthesis

Branched peptide synthesis follows two primary strategies: divergent and convergent.^72^ The divergent approach involves building the peptide directly on the resin, where the branches are added sequentially during synthesis; typically used with hyperbranched peptides and dendrimers such as MAPs.^71^ This method minimises purification steps and facilitates large-scale production but can lead to steric hindrance and reduced coupling efficiency as the branches grow. The convergent approach, in contrast, synthesises the branches independently before attaching them to the core peptide, and is often applied to the synthesis of dendrigraft peptides.^77^ This method allows for the optimisation of individual branches and reduces steric issues but requires additional purification and coupling steps. Advances in orthogonal ligation techniques, such as click chemistry, have made convergent synthesis increasingly accessible and efficient.^58,78,79^ Chemical ligation methods play a vital role in enhancing the complexity and functionality of branched peptides. Techniques such as native chemical ligation (NCL) enable the assembly of large, branched peptides by linking unprotected peptide segments through chemoselective reactions at cysteine residues.^80,81^ Similarly, Cu-catalysed azide–alkyne (CuAAC),^82^ strain-promoted azide–alkyne (SPAAC)^83^ and thiol–ene cycloaddition^84^ have facilitated the rapid and robust attachment of functional groups and peptide branches. Despite these advancements, synthesising branched peptides poses unique challenges. Steric hindrance during the addition of branches can lead to incomplete reactions and lower yields, necessitating the use of excess reagents or extended reaction times.^31,85^ The repeatability challenges associated with synthesising branched peptides and limited side chain chemistries have presented a significant factor in their slow development. Addressing early-stage synthesis issues would significantly advance the field.

**Fig. 3:**
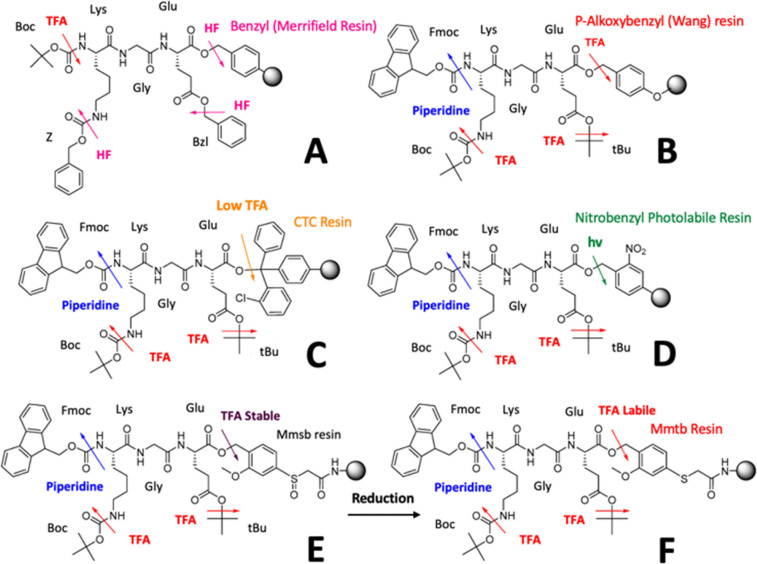
Orthogonal and safety-catch protecting group strategies in solid-phase peptide synthesis. (A) Merrifield strategy using Boc for α-amino protection and Bzl for side chains, both removed by acidolysis but with different kinetics. (B) Bis-orthogonal strategy with Wang linker, Fmoc for α-amino protection, and *t*Bu for side chains, enabling selective deprotection *via* acidolysis or base treatment. (C) Modified bis-orthogonal scheme with Cl-trityl chloride (CTC) resin, allowing peptide release with dilute TFA while retaining *t*Bu-protected side chains. (D) Three-orthogonal approach using a photolabile linker with Fmoc and *t*Bu groups, offering flexibility in deprotection and peptide cleavage. (E) and (F) Safety-catch linkers such as Mmsb, which resist TFA conditions but can be converted into labile forms *via* reductive treatment, providing compatibility with Boc and Fmoc chemistries. Reproduced (adapted) with permission from ref. 91 Copyright 2024, MDPI.

Innovations, such as automated SPPS protocols,^86^ microwave-assisted synthesis,^87^ and improved coupling agents like *O*-(7-azabenzotriazol-1-yl)-*N*,*N*,*N*′,*N*′-tetramethyluronium hexafluor-ophosphate (HATU) and *N*,*N*,*N*′,*N*′-tetramethyl-*O*-(1*H*-benzotriazol-1-yl)uronium hexafluorophosphate (HBTU),^88^ have mitigated these issues by enhancing reaction efficiency and reducing synthesis times. Additionally, the development of advanced resins, such as the 2-chlorotrityl chloride resin,^89^ offers improved swelling properties and stability, further optimising the synthesis of branched peptides (Table 1). Another area of research is the exploration of novel safety catch mechanisms to enable orthogonal deprotection for the implementation of on-resin branching points (Fig. 3). These safety catches can prevent unwanted deprotection, thereby preventing loss of yield.^90^ One notable study converted the side chain protecting groups of six amino acids, three of which can form stable branching points, into a new safety catch system by incubation with Me_3_SiCl-Ph_3_P in tetrahydrofuran. This created labile protecting groups enhancing the control over deprotection steps.^91,92^ Additionally, Zhou^93^ developed a reaction scheme to convert a Boc-protected lysine to a 2,4-dinitro-6-phenyl-benzene sulfenyl (DNPBS) protecting group, which can undergo orthogonal deprotection against Fmoc, contributing significantly to the construction of branched peptide systems with high precision and complexity. These advancements significantly enhance feasibility and scalability, making branched peptide synthesis more accessible (Table 1). By enabling precise control over deprotection steps, researchers can create more complex peptide structures such as cyclic branched peptides or orthogonal functionalisation, addressing some of the longstanding challenges associated with peptide synthesis such as low yield and purity issues.

**Table 1:** Examples of branched peptides currently under development

Name	Type	Sequence	Main properties	Activity	Applications	Ref.
Multiple antigen peptide (MAP)	Dendrimer	(Boc-peptide-Gly_3_)_8_-Lys_4_-Lys_2_-Lys-Ala-OCH_2_	Large multi-arm dendritic structure	Capable of binding to different viral or bacterial antigens	Synthetic vaccine candidate	71, 94 and 95

P-alkyne and P-azide	Hyperbranched	Glu_3_-hexynoic acid or azidohexanoic acid	Dendritic structure functionalised with DNA binding domains	Functioning as a solid phase DNA hybridisation platform	Enhanced COVID-19 detection	96

D3K2 & D3G2	Dendrigraft	Ala-Lys-Lys_3_-(Lys_3_)_2_-(Lys_3_)_4_-(Lys_3_)_8_-(Lys-NH_3_^+^)_8_	Increase permeability through phospholipid bilayer	Transport of genes across the cell membrane	Induce targeted cytotoxicity to cancer cells.	69

HAIYPRH	Dendrimer	PAMAM-PEG-T7	Multi-arm structure containing a gadolinium chelating moiety binding	Able to localise and bind to cancer cells through peptide recognition sequences	Increase magnetic resonance imaging resolution of cancer cells	70 and 97

FD2	Dendrimer	(Fuc-Lys-Pro-Leu)_4_-(Lys-Phe-Lys-Ile)_2_-Lys-His-Ile	Localised binding to *P. aeruginosa* lectin receptors, LecB and LecA	Lectin binding to prevent the deposition of biofilm extra polymeric substance	Treatment the multi-drug-resistant bacteria *P. aeruginosa*	98 and 99

G3KL	Dendrimer	(Lys-Leu)_8_-(Lys-Lys-Leu)_4_-(Lys-Lys-Leu)_2_-Lys-Lys-Leu	Terminal positivity charged amino acids	Disrupt bacterial cell membranes, leading to cell death	Combating anti-microbial resistance	100–102

(LDLK)_3_	Dendrigraft	(Leu-Asp-Leu-Lys)_3_	Self-assembly branched peptide nanostructure	Utilising a lysine branching point as a cross-linking agent between three linear peptides	Enhanced hydrogel stiffness using branching points to create a more versatile SAP	30

Denpol	Dendrigraft	Cys-Cys-Lys(Lys(Lys_2_))-Cys-Cys-Lys-(Lys(Lys_2_))	Combinatory pH responsive, hydrophilic/phobic branched structure	Permeability through cell membrane to deliver siRNA	Alternative non-viral siRNA delivery vector	75

## 3. Controlling the physical properties of branched peptides

### 3.1 Customisation of branched peptides for biomedical applications

**Fig. 4:**
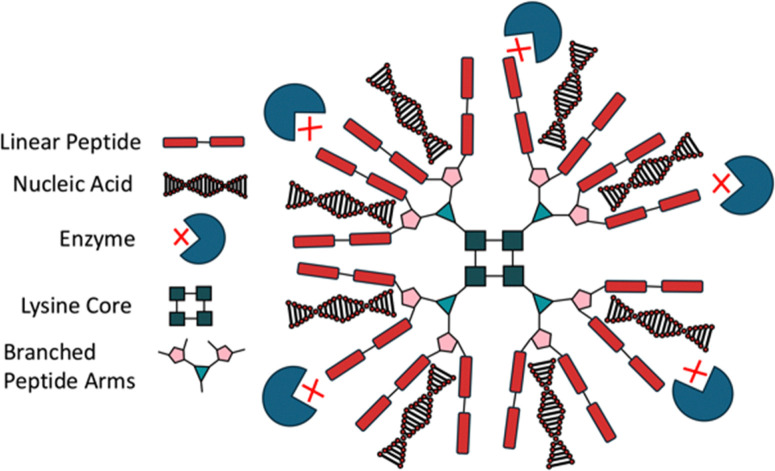
A dendrimer polylysine star. Of the three branched peptide types, dendrimers are typically used for drug delivery. Generating a lysine core can increase the production of available coupling points. In this case, a lysine core, depicted by the central squares, has created coupling points for red rectangle linear peptides. The densely packed structure and coupling of adhesive peptides (red rectangles) allows for the embedding of cargo such as nucleic acids (red & black triple helices) affording protection from enzymatic attack (outer blue circles). This allows for safe and practical transport of molecular mediators such as small-molecules and plasmids to the target cell.

Customisation of branched peptides is critical for developing functional materials tailored to applications such as drug delivery, tissue engineering, and ECM mimics. Customisation can include pH dependent degradation, modulation of material stiffness, or the incorporation of cyclic peptides or non-natural amino acids. Branched peptides offer unique advantages over linear peptides due to their multivalency, tuneable architecture, and enhanced stability. For instance, drug carriers leveraging branched peptide frameworks benefit from increased drug binding efficiency and controlled release kinetics.^103^ Tryptophan-rich peptide dendrimers (TRPDs) utilise lysine branching with tryptophan-terminated arms, improving drug stability and reducing degradation by enhancing interactions such as hydrophobic, π–π stacking, and hydrogen bonding, making them effective vehicles for gene therapies and DNA-targeting drugs (Fig. 4).^104,105^ Similar to TRPDs, dendrimers are also customisable, by changing the terminal amino acids and therefore changing the properties of the material. Mixson and colleagues added a histidine-rich tail to their branched dendrimer, discovering that the new design improved *in vitro* gene transfection efficiency.^106^ Similarly, poly(amidoamine) (PAMAM) dendrimers, using a lysine core, offer a high degree of customisability,^107^ having been functionalised with dodecapeptides to deliver interleukin-12 plasmids to mesenchymal stem cells, significantly improving the drug's cancer-targeting ability.^33^ Others have used un-natural amino acid derivatives to provide branching points, such as 2,3-diaminopropionic acid, which was used to develop a three-arm dative chelator for Ca^2+^ binding and transport.^108^ Branched peptides can also be designed to mimic the structures of other biological molecules, such as phospholipids, *e.g.* by using the terminal lysine as the polar head, and histidine/leucine as the non-polar body, to increase cell membrane transfection through the amphipathic structure.^109^ This versatility in designing branched peptides not only enables fine-tuning of their structural and functional properties but also opens avenues for combining them with other peptide systems, such as cyclic peptides, to further enhance their biomedical potential.

**Fig. 5:**
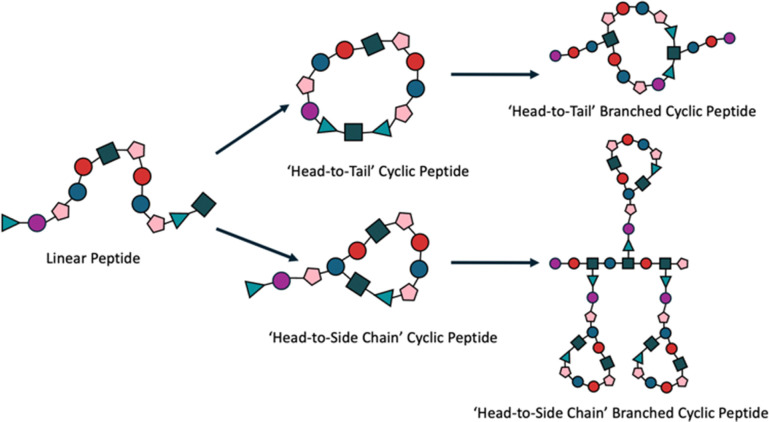
Generation and incorporation of cyclic peptides into branched peptide systems. The process begins with a linear peptide precursor, which can be cyclised into two distinct types of cyclic peptides: ‘head-to-side chain’ cyclic peptides, characterised by a cyclical end with a linear tail, and ‘head-to-tail’ cyclic peptides, featuring a simple closed-ring configuration. These cyclic peptides are subsequently incorporated as branching points into branched peptide systems, enhancing the versatility and stability of the resulting biomaterials.

Cyclic peptides, characterised by the circular presentation of the amino acid sequence, either across the entire peptide or at the termini, offer distinct advantages in biomedical applications due to their conformational rigidity and enhanced proteolytic stability (Fig. 5).^110,111^ These features improve target binding affinity and selectivity while reducing cellular toxicity, making them promising candidates for therapeutic use.^112,113^ Their stability is underscored by the 18 cyclic peptides currently approved for clinical trials.^113^ When integrated with branched peptides, the resulting hybrid systems exhibit enhanced functionality, combining the stability and specificity of cyclic peptides with the multivalency and tunability of branched architectures.^114^ A practical demonstration of this synergy was provided by Lu *et al.*, who coupled a cyclic peptide to the aspartic acid carboxyl group of a branching peptide, facilitating *in situ* ligation with an N-terminal cysteine.^115^ The resulting cyclic-branched peptide was synthesised with a yield of 50%, showcasing a novel approach to creating complex peptide structures. Such hybrid systems exemplify how customisation can generate multifunctional biomaterials, with potential applications in drug delivery, targeted therapeutics, and regenerative medicine. The iterative ‘denpol’ strategy developed by Zeng *et al.* further highlights this potential, demonstrating the construction of highly branched macromolecules through dendritic polypeptide coupling.^75^ This approach enables fine control over structural features, creating materials tailored for specific biomedical functions. Integrating cyclic motifs with branched systems also aligns with broader efforts to develop multi-receptor binding peptides, exemplified by polymer systems such as hyaluronic acid and *N*-(2-hydroxypropyl)methacrylamide, which are currently being trialled for similar applications.^116,117^ The ability to customise these hybrid structures further enhances their relevance, offering routes to creating tailored therapeutics with improved efficacy and specificity.

### 3.2 Control of degradation rates

**Fig. 6:**
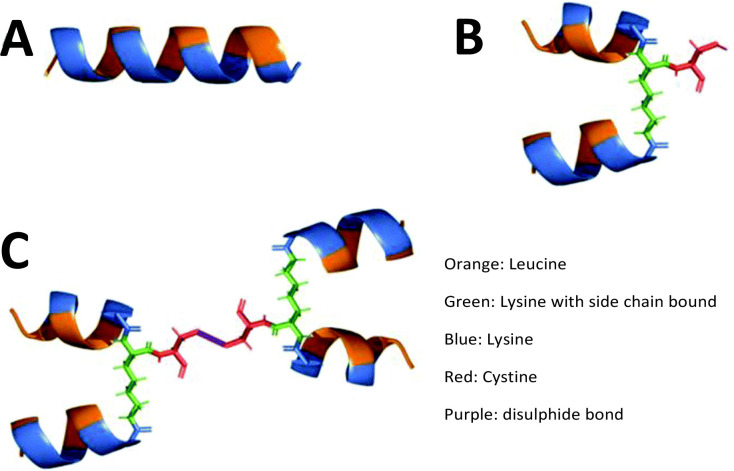
Structural representations of linear and branched α-helical peptides. Schematic illustrations showcasing variations in peptide architecture. (A) The linear (LLKK)_4_ peptide with a single continuous sequence. (B) A 2-arm branched peptide, [(LLKK)_2_]_2_κC, with branching introduced *via* a lysine residue. (C) A 4-arm branched peptide, {[(LLKK)_2_]_2_κC}_2_, featuring a more complex branched structure derived from multiple lysine-mediated branching points. These designs highlight the increasing structural complexity and potential functional versatility of α-helical peptides with branching. Reproduced (adapted) with permission from ref. 122 Copyright 2024, Royal Society of Chemistry.

Controlling the degradation rate of peptide-based structures has been highlighted throughout this manuscript. Various strategies have been employed to reduce peptide degradation and minimise premature loss of function while maintaining biological activity. Common strategies include the addition of motifs which cannot easily be cleaved by proteolytic enzymes, such as cyclisation,^118^ incorporation of d-amino acids,^119^ or the use of unnatural amino acids.^120^ These methods enhance resistance to degradation, increasing the material's versatility, which is crucial for *in vivo* applications such as cancer immunotherapies^121^ and biomaterial-based tissue engineering.^122^ Cross-linking is another method typically used to reduce degradation rates. However, in selecting crosslinking methods we must also consider the end application to avoid cytotoxicity. It is here that the introduction of branching points can create new opportunities to develop complex matrices without the need for potentially toxic crosslinking agents whilst benefiting from reduced degradation rates and an improved active half-life. This is demonstrated by Wang,^123^ who combined an α-helical peptide with a cystine lysine dendrimer, inducing cancer cell degradation (Fig. 6). They found that using a branched peptide was more effective than using the linear counterpart and reduced the degradation rate, proving a viable potential cancer therapy.^123^ The increased half-life of the peptide compared to the linear counterpart provides a suitable design template that could be applied to other peptide-based drug targets such as glucagon-like peptide-1 & 2 receptors in the treatment of type 2 diabetes,^124–126^ gonadotrophin-releasing hormone receptor as a novel drug candidate for prostate cancer,^127^ and parathyroid hormone-related protein receptor involved in osteoporosis therapies.^128^ By understanding and manipulating the degradation profile of peptide materials, we can better mimic the biological environment and improve clinical outcomes.

As previously discussed, branched peptide systems are typically synthesised and assessed for their structural and biomimetic characteristics.^32,46,47^ However, research on the physical properties *in vitro*, such as degradation rates, is rarely assessed. One of the few examples where *in vitro* degradation studies have been employed is shown by Agazzi,^129^ where the branching structure was used to create pH- and enzyme-labile degradation profiles. This work highlights the potential of branched peptide structures to enhance biomaterial stability, thereby improving future material designs. Exploring this research area is crucial as it can reveal how branch points on peptides can enable greater stability and improve the longevity of biomaterials. It could also provide valuable data on material half-life when exposed to typical matrix proteases. Peptide degradation can be assessed from macro and molecular perspectives. At the macro level, rheological studies allow measurement of the viscoelastic properties of peptide-based solutions or soft solids, such as elastic (*G*′) and viscous (*G*′′) moduli. These measurements help in understanding the changes in the mechanical properties of peptides. Optical techniques, such as turbidity or light scattering, can also monitor bulk structural degradation.^130,131^ At the molecular level, the combination of high-performance liquid chromatography (HPLC)^132^ and mass spectrometry (MS)^133^ can separate, quantify, and identify degradation products or intact peptides. Spectroscopic methods such as circular dichroism (CD)^134^ track structural changes such as the formation or loss of secondary structures, α-helices and β-sheets. These methods together offer insights into the stability and breakdown of peptide-based materials. Such information is directly relevant to material usability and marketability since a biomaterial that degrades too quickly before achieving its therapeutic potential holds limited clinical value. Pharmacokinetic studies routinely assess degradation rates to determine the therapeutic effectiveness of drug candidates.^135,136^ These studies provide a deeper understanding of retention times and degradation rates, which are critical for predicting the *in vivo* behaviour of the therapeutic agents. Applying a similar rationale to branched peptides could help ascertain their *in vitro* degradation rates and ultimately lead to the development of more clinically relevant materials. This approach would ensure that branched peptide-based biomaterials maintain their functional integrity long enough to exert their intended therapeutic effects. This dual focus on structural integrity and material longevity is exemplified by innovations such as the lysine knot (discussed later), which enhances peptide stability and stiffness to better suit biological environments.

### 3.3 The impact of branching on the macromolecular structure

**Fig. 7:**
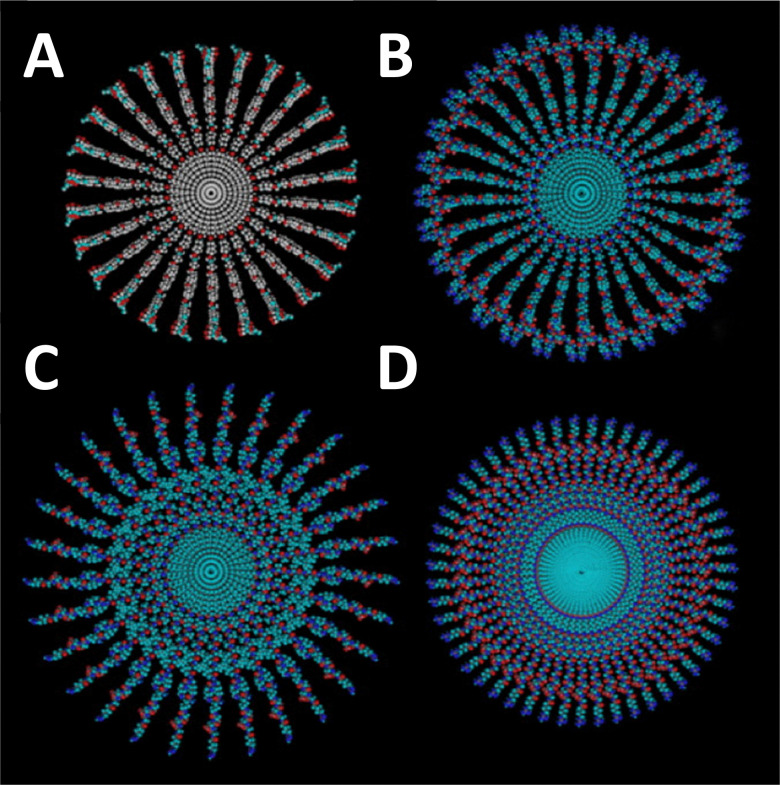
Morphological differences in peptide amphiphile (PA) nanofibers formed from branched and linear PAs. Cross-sectional representations of nanofibers highlighting structural variations based on the PA architecture. (A) Nanofibers derived from a branched PA featuring a single cyclic RGDS epitope. (B) Branched PA nanofibers incorporating two RGDS epitopes. (C) Nanofibers formed by a branched PA with a single RGDS epitope. (D) Linear PA nanofibers containing a single RGD epitope. These variations demonstrate how epitope arrangement and PA structure influences nanofiber morphology and organisation. Reproduced with permission from ref. 7 Copyright 2024, Elsevier.

Utilising branched peptides to enhance secondary supramolecular structure is not a novel concept. A 2003 study on ‘T-SAFs’ introduced a branched helical coiled-coil peptide, exhibiting self-assembly branched nanofiber formations.^137^ Similar studies later followed, creating a branched peptide amphiphile which self-assembles into nanofibrils, for fibre-bonded poly(glycolic acid) scaffolds^138^ and RGDS epitope presentation.^139^ Building on the concept of using branching points to influence nanofiber formation, this work was continued in 2007, where branching extended the diameter of the nanofibers and reduced mechanical stiffness (Fig. 7).^7^ This was later adapted to guide neural cell network development, linked to the reduced stiffness of wider, branched, fibrils compared with linear fibres.^32^ The system developed by Sur and colleagues utilised two β-sheet peptide amphiphiles, one linear and one branched, with varying stiffnesses, between 7.3 kPa and 22.9 kPa, to guide fibroblast and neuron development. When assessed using 3T3 fibroblasts and mouse hippocampal neurons, they found different growth patterns between the materials, owing to the cellular preference of material stiffness. However, other branched peptide designs attempt to use branching points as a peptide-bond-based crosslinking strategy, enhancing mechanical strength.^30^

The lysine knot, developed by Pugliese, uses a Nε-di-Fmoc-lysine to bond three linear peptides simultaneously.^30^ Rheological studies showed that the linear peptides displayed weak associations, creating a loose network and a soft material. In contrast, when mixed with the lysine knot at a high molar ratio, the stiffness increased by approx. 100-Fold, with a final stress tolerance of 8.14 kPa.^30^ These studies collectively show how branch peptides can be customised to both reduce or enhance stiffness depending on the design. Such stiffness changes can have a significant impact on cell behaviour. For reference, fibroblasts exhibit a stiffness of ∼3 kPa, whereas lung tissue is ∼0.2 kPa, contrasted with the stiffness of bone at just under 200 000 kPa.^140^ Several other studies detail how cells can transduce mechanical forces into biological signals, providing the basis for directing cell behaviour based on stiffness.^141–143^ Utilising cell receptors such as RHO-GTPase (guanosine triphosphatase)^144^ and transducers, including YAP (yes-associated protein) and TAZ (tafazin-associated protein),^145^ the ability to control a material's stiffness creates a more versatile 3D system that can be adapted to different tissue and cell types depending on the clinical need. By considering both the 3D environment as well as the stiffness, supramolecular designs could revolutionise other research fields such as organ-on-chip and organoid development. Many existing platforms in these areas do not offer modifiable stiffnesses that prevent natural gene expression^146^ with specific spatiotemporal mechanical properties.^147,148^ Therefore, innovations in peptide design, and the incorporation of branching points to fine-tune mechanical properties and degradation rates, are key to overcoming these challenges and advancing the development of realistic and functional organ-on-chip and organoid systems.

## 4. Biomimetic functionalisation

### 4.1 Bioactive peptide functionalisation

By incorporating bioactive sequences, branched peptides can further enhance cell signalling leading to improved cell adhesion and differentiation, making them more effective than inert materials for drug delivery, tissue engineering, and regenerative medicine. From earlier discussions, it is unsurprising that several researchers have used RGDS to functionalise their initial branched peptide designs. Combining the biomimetic functionality of RGDS with multivalent branched peptides is demonstrated by Liu who synthesised a linear (RGD)_3_ peptide functionalised with two cyclic RGD peptides, creating two dendrigraft branched peptides.^149^ As discussed earlier the use of cyclic peptides has many physical advantages, but when used in conjunction with functional peptide moieties they can represent the *in vivo* environment more closely, resulting in an increased upregulation of cell proliferation through enhanced cellular attachment.^150,151^ Similarly, others have used an IKVAV-capped dendrimer embedded into a cross-linked collagen hydrogel which selectively upregulated rat Schwann cell proliferation and reduced human dermal fibroblast cell proliferation.^152^ These approaches demonstrate the versatility and efficacy of incorporating bioactive peptide sequences into biomaterials to achieve specific cellular responses.

Functionalisation strategies have since developed from single epitope peptides to multiple epitopes, taking advantage of the availability of serval binding sites typically seen with branched peptide termini. The use of multiple peptide epitopes enhances cell signalling by simultaneously activating several integrin receptors, leading to a more robust cellular response.^153^ This approach better mimics the complex biochemical environment of native tissues, which often presents multiple binding sites to cells.^154^ Mas-Moruno utilised a lysine branching point to display RGDS and PHSRN simultaneously to promote integrin binding to metallic implants.^31^ Whilst bound to a titanium plate, the dual-functionalised system induced significantly increased cellular spreading and proliferation compared to non-coated surfaces or materials functionalised with a single peptide variant. Others have followed similar strategies to introduce more than one integrin-interacting peptide sequence to their own materials. For example, RGDS and IKVAV were presented simultaneously on another titanium implant, influencing cellular adhesion, proliferation, viability, and angiogenesis of endothelial cells.^132^ Dual-functionalised systems could reduce the risk of implant rejection and enhance integration with surrounding tissue, as seen with RGD and GFOGER.^9^ Furthermore, by selecting appropriate combinations of peptide epitopes to target desired cellular responses, this strategy allows for the customisation of biomaterials for specific applications.^155^

### 4.2 Hybrid-polymer functionalisation of branched peptides

**Fig. 8:**
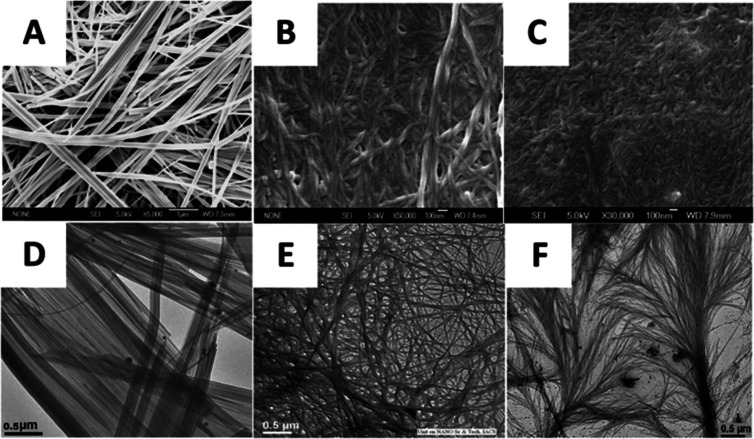
Self-assembling branched peptide organogels. Three different generations of branched peptides were compared, each undergoing the coupling of an additional generation of amino acids. (A)–(C) Field emission scanning electron micrographs of the resulting gels at the three different generations. (A) Shows the fewest number of generations and least dense network, whilst (B) is the median number of generations and density, and (C) is the largest number of generations forming the densest network. (D)–(F) High-resolution transmission electron micrographs (HR-TEM) of the branched gels obtained from the different generations of dendritic 1,2-dichlorobenzene. Reproduced with permission from ref. 162 Copyright 2024, American Chemical Society.

Peptide-based functionalisation has been shown to increase bioactivity or provide sites of attachment for other cell-interacting chemistries, such as cell receptors or non-polar molecules. Although there are many examples of peptide functionalisation, less attention is focused on using other natural polymers to functionalise linear or branched peptide systems. In the body proteins readily undergo polymer functionalisation to enhance protein stability and secondary folding,^156^ reduce proteolytic degradation,^157^ and facilitate intra-/extra-cellular transport.^158^ Using a similar approach, bioactive polymers can be coupled to peptide-based materials to improve biological properties, which is not easily achieved through peptide chemistry, or the addition of small moieties, alone. Lipidated proteins, integral to native healthy tissues, matrices, and cells, facilitate metabolic transport,^159^ cell membrane anchoring,^160^ receptor binding,^159^ signal transduction,^161^ and intracellular trafficking^162^ by providing a means to non-polar interactions. Whilst this does include some non-polar amino acids such as alanine, valine, and leucine, they cannot achieve the same non-polar interactions as lipid molecules, owing to their natural amphipathic design. Combining the polar nature of peptides, multi-valency and the customisation of branched peptide designs, with the non-polarity of lipid molecules presents an opportunity to create dynamic biomaterials capable of interacting with multiple cell types, membranes and receptors. This was achieved in 2010, where researchers used a dendritic branching peptide with an aspartic acid core to create a self-assembling organogel which formed in various organic solvents at low concentrations^163^ (Fig. 8). Similarly, Haridas synthesised an asymmetrical branched peptide dendrimer using lysine, aspartic acid and glutamic acid branches, coupled to dodecylamine to create the lipidated compound.^164^ Due to the asymmetry of the design, the peptides self-assembled in both aqueous and organic solvents. Whilst being able to utilise organic solvents, organogels can sequester non-polar drugs and administer through direct cell membrane interaction, allowing for targeted drug delivery,^165^ and allowing access to cellular receptors otherwise inaccessible to polar peptides. This was also demonstrated by Sivades, who showed that lipidated glutamic acid dendrimers increased the chondrogenic differentiation of adipose-derived mesenchymal stem cells, with one lipopeptide regulating collagen type 1 production, suggesting potential for hyaline cartilage formation and prevention of hypertrophic development.^166^ While further research in *in vitro* models is necessary to fully understand the impact of these branched peptide hybrids, these findings indicate that providing interactions with non-polar molecules could aid drug delivery^167^ as well as an upregulation in ECM fibre formation which would otherwise have not been possible without lipidation.

Other natural polymers, such as carbohydrates also pose a significant benefit to branched peptides. Glycopeptides are used frequently by the body to promote cell–cell recognition,^168^ signalling,^169^ and to regulate the immune response.^170^ They facilitate interactions with lectins,^171^ and other carbohydrate-binding proteins,^172^ thereby influencing various physiological and pathological processes. When combined with biomaterials they can offer unique advantages as seen in next-generation antibiotics such as vancomycin^173^ and teicoplanin,^174^ anticancer drugs such as bleomycin^175^ and more recently supramolecular biomaterials.^176,177^ These developments have led to materials which react to pH,^178–180^ redox,^181,182^ and temperature.^183,184^ To this end, branched peptide systems have been further modified to adopt glycans, *e.g.*, Li used a triazine core with three glycosylated phenylalanine residues bound in a ‘Y’ shaped conformation.^185^ After 24 hours of culturing with RAW264.7 macrophages, the studies showed that there was no evidence of cytotoxicity-driven cell death, though pro-inflammatory cytokines were observed. The presence of pro-inflammatory cytokines does suggest M1 macrophage polarisation, is not suited to ECM regeneration, although further studies would be needed to confirm. Other researchers have modified linear peptides for a variety of functions such as HIV inhibition^186^ and to cross the blood–brain barrier,^187^ as well as structural enhancements including *in vivo* peptide stability^188^ and half-life.^189^ Again, studies in this area remain limited and further research is needed to fully reap the benefits of branched peptide glycosylation as seen with linear peptides and to further understand, and exploit, their potential for biomedical applications. Of note is the need for comprehensive *in vivo* studies essential for the evaluation of their pharmacokinetic characteristics, biodistribution, and long-term effects. As these advances are realised, glycosylated branched peptides could revolutionise drug delivery, enhance therapeutic specificity, and contribute to innovations in immune regulation^190^ and interactions with glycan receptors including those involved in diabetes.^191^ With more than 90% of ECM proteins being glycosylated, this increases the mimetic qualities of ECM-inspired biomaterials.^192^

## 5. Medical & clinical application of branched peptides

The unique structural attributes of branched peptides, discussed throughout this review, including their multivalency^103^ and enhanced stability,^30^ have positioned them as promising candidates for medical and clinical applications. This is particularly evident within oncology and infectious diseases. Their modular nature allows for targeted interactions,^104^ precise delivery,^33^ with minimal immunogenicity.^193^ In oncology, branched peptides have shown considerable potential as targeted drug-delivery vehicles and anticancer agents. By leveraging multivalent interactions, branched peptides can selectively bind to overexpressed tumour receptors. Research in this area is primary focused on sulphated glycosaminoglycans (GAGs) and G protein-coupled receptors using the tetra-branched peptide human neurotensin (NT4).^194–196^ NT4 selectively binds to the NTS1 receptor overexpressed on certain malignant tumours,^197^ and was modified in 2011, creating a tetra-branched version. Comparatively, the branched version was found to increase the cytotoxicity of the drug doxorubicin, through increased binding affinity and tumour selectivity.^198^ This system has since been tested to compare linear and branched peptides,^199^ explore the role of heparin-sulphate proteoglycans receptors,^194^ and to create new derivatives with selective binding and *in vitro* cytotoxicity.^200^ The versatility and biocompatibility of branched peptides have also been used to increase the solubility of drugs, to preferentially accumulate in tumour tissues, and improve drug delivery efficiency. In one such example two branched amphiphilic peptides were self-assembled into nanofibrils, loaded with anti-cancer drugs, allowing for selective and prolonged drug release with increased cytotoxicity to cancer cells.^201^ Separately, branched peptides have been created with anti-cancer properties without requiring additional drug doping. Recncinai and colleagues developed two branched peptides, BOP7 and BOP9, which selectively bind to cancer cells, causing the release of damage-associated molecular patterns. This led to immunogenic cell death, equating to a 20% inhibition of tumour growth and grafting.^202^

Other clinical applications of branched peptides include the treatment and reduction of microbial disease and infection. While antimicrobial resistance (AMR) continues to present considerable challenges, there is research on the potential use of peptides as therapeutic agents to act as front-line drugs to fight AMR.^203^ James Tam in 1988^71^ first introduced this potential through the development of the earlier-mentioned MAPs as vaccine candidates. By 1999, the use of MAPs as antimicrobial agents was assessed, expressing eight copies of the antimicrobial peptide lactoferricin, and was noted to have had significant antimicrobial effects even on methicillin-resistant *Staphylococcus aureus*.^95^ More recently, antimicrobial peptide dendrimers (AMPDs) have been developed to interact with microbial membranes through multivalent binding, leading to membrane disruption and microbial death, with several reviews detailing their successes.^73,204^ Compared to linear peptides, branched AMPDs exhibit increased potency,^100,205^ reduced susceptibility to proteolytic degradation,^206^ and present scope for more complex designs.^207^ It has also been noted that bacteria are much slower to develop resistance against AMPDs once deployed *in vitro*.^208–210^ Biofilms present further complications, acting as a physical barrier against anti-microbial agents.^211^ In 2016 researchers developed a repeating Arg-Trp branched peptide (2D-24) which was shown to eradicate up to 94% of biofilm-producing multi-drug-resistant *P. aeruginosa* at concentrations that are not toxic to mammalian cells.^193^ Despite these early studies presenting clear potential for branched peptides to make significant impact on global health issues their progression from bench to bedside remains challenging.

Currently, only a handful of branched peptide-based therapies are in industrial development or have reached the market, such as the AMPD, VivaGel, which has been approved for clinical use in the United Kingdom and Australia for the treatment of bacterial vaginosis.^212,213^ AMPDs appear to show promise in moving from the laboratory to the clinic quicker than other branched peptide constructs with more work going into *in vitro* toxicity studies.^214^ A recent paper identified several tetra-branched AMPDs and tested these at 10 times the minimum inhibitory concentration and found that most showed no cytotoxicity against red blood cells.^214^ Although this is a limited view of cytotoxicity compared with *in vivo* studies, it does provide advancements in the field. Similarly, after their development in the 1980s, some MAP designs have been used for diagnostic tests^215^ or biosensors,^216^ but very few have reached clinical trials and those that did failed due to a low immunogenic response.^217^ Some more recent *in vivo* assessments have produced constructs that elicit promising immune responses, though these have yet to be approved for clinical trials.^94,218^ Thus, the popularity of MAPs appears to have decreased after clinical trial failure, with a decline in pharmaceutical interest leading to a lack of overall progression. Key to this lack of interest is addressing the limitations, including scalability of synthesis,^219^ high production costs,^220^ and concerns about long-term biocompatibility.^195^ Furthermore, many branched peptide therapeutics in the pipeline face regulatory hurdles related to their structural complexity and lack of precedent in clinical use.^221^ The few approved branched peptide systems highlight the need for further innovation to address these shortcomings. For instance, while their multivalency enhances drug delivery and target specificity, it may also increase the risk of off-target interactions or unexpected immunogenicity.^195,222^ These limitations underscore the importance of continued research into optimising the design and synthesis of branched peptides to balance efficacy, safety, and cost-effectiveness. By overcoming these challenges, branched peptides have the potential to redefine clinical therapeutics, offering novel strategies to address unmet clinical needs and paving the way for their adoption in clinical practice.

## 6. Other applications of branched peptides

**Fig. 9:**
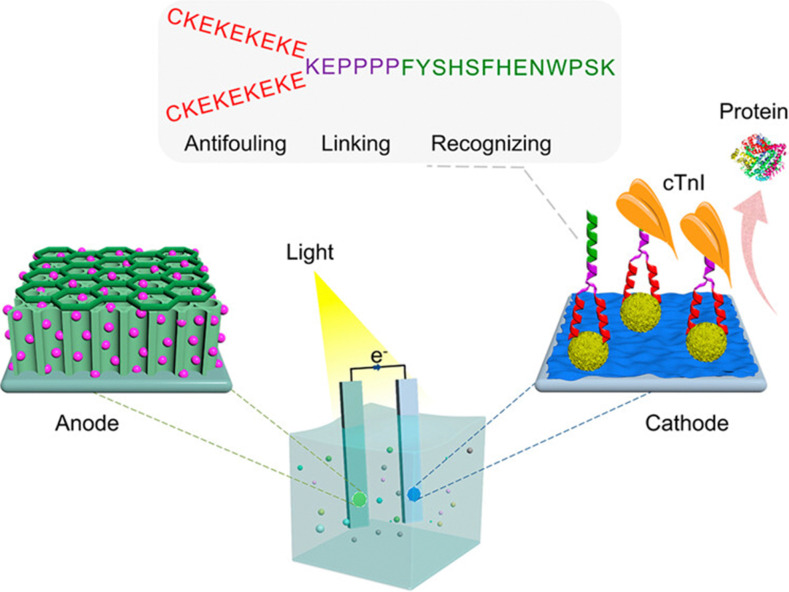
Branched peptide photoelectrochemical biosensor design. The photoelectrochemical biosensor integrates a novel engineered branching peptide (EBP) into a dual-photoelectrode system. The EBP, has a Y-shaped configuration featuring a recognition backbone and two antifouling branches. The biosensor demonstrated a high photocurrent response and strong antioxidation properties with excellent sensitivity, specificity, and anti-interference in detecting the cardiac troponin I (cTnI) biomarker. Reproduced with permission from ref. 222 Copyright 2024, American Chemical Society.

In addition to their cell-promoting and tissue-integrating biomimetic qualities, branched peptides are being assessed for other applications including biosensors,^223^ 3D printable bio-inks^224^ and antimicrobial drug candidates.^225^ However, similar to the aforementioned applications, the ability of branched peptides to imitate native chemical motifs remains key to creating these new platforms which interact with, and influence, molecular biology. An example of this is the novel photoelectrochemical biosensor developed for detecting cardiac troponin I in human serum. This system features a uniquely engineered branching peptide with enhanced antifouling properties^223^ (Fig. 9). Integrating a dual-photoelectrode system (C_3_N_4_/TiO_2_ photoanode and AuPt/PANI photocathode), the Y-shaped dendrimer branched peptide offers superior antifouling and recognition capabilities compared to linear peptides. The biosensor demonstrated high sensitivity, specificity, and anti-interference for detecting cardiac troponin I (cTnI). Similarly, Li combined a zwitterionic antifouling peptide and an antibacterial peptide with a recognition peptide aptamer^226^ to produce an electrochemical biosensor for detecting the receptor binding domain of the SARS-CoV-2 spike protein. Other uses for branched peptide-based biosensors include antibody mimics,^227^ clinical sample testing,^228^ antibody detection,^229^ cellular quantification^230^ and nucleic acid detection.^96,231^

In recent years, companies such as CELLINK™ and 3DBio™ have developed the technology to create cell-embedded 3D biomaterials to produce tissues such as cardiovascular,^232^ bladder,^233^ and bone.^234^ A study on bioinks used a reinforced peptide-dendrimer^224^ combining a peptide-dendrimer branched PEG with end-grafted norbornene (PDN) and cysteamine-modified HA (HC), enhancing crosslinking. The combined HC-PDN showed improved mechanical and rheological properties, reduced reactive oxygen species accumulation, and enabled bioprinting of complex structures with high shape fidelity, such as a hepatic tissue model with HepG2-C3As, LX-2s, and EA.hy.926s. As described earlier, branched peptides are ideally suited to the generation of customisable 3D structures, with tailored degradation rates^129^ and stiffnesses,^32^ and for the incorporation of functional motifs such as RGDS.^149^ These features also allow branched peptides to be explored as potential candidates for complex 3D bioinks towards the generation of organoids and organ-on-chip models, to model disease progression,^235^ tumour growth,^236^ organ replacements,^237^ and others.^238^

By combining rapid production technological advancements with bottom-up peptide design strategies we are afforded limitless opportunities to explore healthcare applications that extend far beyond the tissue repair and regeneration need described at the beginning of this review. There are opportunities for growth in all the areas described and, as such, we are likely to see significant advances in the coming years, beyond those described here.

## 7. Conclusions

The exploration of branched peptide-based biomaterials represents a significant leap forward in biomedical research, inspired by nature's own designs. Enhanced by breakthroughs in SPPS, such as safety catches and novel amino acid side chain protecting groups, the precision, yield, and scalability of synthesis have all dramatically improved, presenting new opportunities for branched peptide research. Comprising three main categories—hyperbranched, dendrimer, and dendrigraft—branched peptides form a range of nanostructures and are easily tuneable for different applications including drug delivery, bioimaging, and tissue engineering. Advancements in the control of degradation and stiffness profiles of these designs have in particular led to materials that are capable of self-assembling into higher-ordered 3D structures. Additionally, the ability to functionalise branched peptides with short motifs such as RGDS, IKVAV, and PHSRN, as well as non-peptide polymers such as lipids and carbohydrates, facilitates physical, molecular, and cellular interactions that were previously thought to be unattainable *via* traditional synthetic material design.

While challenges such as scalable syntheses, comprehensive degradation rates from *in vivo* studies, and the limited *in vivo* research in the area, still exist the progress made thus far, combined with technological advancements, indicates a bright future for branched peptide design, holding the promise of innovative therapeutic strategies and a transformative impact on biomedical applications in future.

## Author contributions

Nazia Mehrban and Jody M. Mason were responsible for the conceptualisation and revision of the article. Matthew J. Little was responsible for the investigation, visualisation, drafting of the manuscript, and the production of all figures used in the article.

## Data availability

No primary or new data was collected for the purposes of this article, nor was any software, code or programs used to generate any of the information displayed in this article.

## Conflicts of interest

All Authors declare no conflict of interest.
